# Changes in prostaglandin E2 in patients with idiopathic overactive bladder syndrome after botulinum toxin type A treatment: is there a clinical benefit?

**DOI:** 10.1186/1471-2490-14-85

**Published:** 2014-11-04

**Authors:** Axel Hegele, Sonja Knippschild, Carsten Frohme, Jörg Hänze, Peter Olbert, Rainer Hofmann

**Affiliations:** Department of Urology and Pediatric Urology, Philipps University Marburg, Medical School, Marburg, Germany

**Keywords:** Prostaglandin E2, Overactive bladder, Biomarker, Botulinum toxin type-A

## Abstract

**Background:**

The causality of overactive bladder syndrome (OAB) is still not fully understood. Several studies indicate a significant increase of prostaglandin E2 (PGE2) in patients with OAB. However, in order to clarify whether these compounds can help to objectify the clinical diagnosis, further studies are needed. This prospective study aims to analyze PGE2 blood levels (sPGE2) in patients with OAB before and after botulinum toxin type A (BoNT-A) therapy.

**Methods:**

Blood samples were obtained from 56 patients (52y, 18–87) with idiopathic OAB. sPGE2 levels were measured before and 4 weeks after BoNT-A treatment by enzyme linked immunosorbent assay (ELISA). 31 healthy persons with normal bladder function served as control group (59 y, 21–72). sPGE2 was set in relation to clinical data and the severity of OAB (wet/dry). The statistical data analysis was performed by using the non-parametric Mann–Whitney U test and paired t-test.

**Results:**

Significant higher sPGE2 levels were detected in patients with OAB compared to members of the control group (2750 pg/ml vs. 1674 pg/ml, p < 0.005). Furthermore sPGE2 levels were increased in patients with OAB wet compared to OAB dry (p <0.01). In 30 patients sPGE2 levels decreased significantly after BoNT-A treatment compared to baseline (2995 pg/ml vs. 1486 pg/ml, p <0.005). Patients reported an average drug effect of 9 month (0–19); incontinence pads were needed significantly less frequent (p < 0.05). 3 patients reported no postoperative effect. sPGE2 increased in two patients compared to initial levels, a single patient showed a remotely decreased sPGE2. Six patients were treated repeatedly with BoNT-A after showing an sPGE2 re-rise.

**Conclusions:**

sPGE2-level is increased in patients with OAB. We could prove a significant decrease of sPGE2 after BoNT-A treatment. In this small cohort we could demonstrate a correlation between OAB and sPGE2, especially in the non-responder group. The use of sPGE2 as a biomarker in diagnostics and follow-up after therapy seems promising. To what extent sPGE2 can be useful as such needs to be examined prospectively in a larger population.

## Background

The prevalence of idiopathic overactive bladder syndrome (OAB) in Europe is estimated at 16% in men and women [1]. Sixty-eight percent of the women and 60% of the men are bothered by OAB. It is a serious problem that negatively affects the quality of life [1, 2]. According to the International Continence Society (ICS), OAB syndrome is defined as urinary urgency usually accompanied by frequency and nocturia, with or without urge incontinence, in the absence of causative infection or pathological conditions [3]. The reasons for these symptoms are still unknown, although already many studies could uncover compounds and pathways inducing detrusor contractions and/or detrusor over activity (DO) [4–6].

One of these compounds originates from arachidonic acid. Prostaglandin E2 (PGE2) is a cytoprotective eicosanoid, which is synthesized de novo from the detrusor muscle or urothelium [7]. It has been shown that PGE2 is increased in carcinogenesis, urinary tract infections and overactive bladder syndrome [8, 9]. The urinary PGE2 level is significantly increased in OAB and correlates negatively with the maximum cystometric capacity [10]. In rats, intravesical instillation of PGE2 triggers detrusor contractions, but topical application to the urethra leads to urethral relaxation in rats [11]. Bladder storage dysfunction and elevated urinary PGE2 levels have been proven to be enhanced in rats after causing severe damage to the urothelial barrier [12].

Once a patient is diagnosed with OAB syndrome, lifestyle modifications and bladder retraining is suggested. Pharmacological treatments are needed to improve quality of life if behavioural training fails. Due to many side effects the majority of patients discontinue anticholinergic medication [13]. Botulinum toxin (BTX) is a potent neurotoxin, used to treat different pathologies such as blepharospasm or spastic torticollis. It attacks one of the fusion proteins (SNAP-25, syntaxin or synaptobrevin) at the neuromuscular junction, preventing vesicles from anchoring to the membrane to release acetylcholine. By inhibiting acetylcholine release, the toxin interferes with nerve impulses and causes flaccid paralysis of muscles [14].

BTX type A (BoNT-A) and Onabotulinum toxin are used to treat neurogenic and/or overactive bladder when oral anticholinergic medication is not successful or not tolerated.

Injected into the detrusor muscle, it reduces urgency and frequency and improves quality of life. Common but rare side effects are urinary retention and urinary tract infections [15–17].

Cause and development of OAB are still unknown, thus further studies are needed to enhance diagnosis of OAB as well as to identify markers useful for daily clinical routine in verifying the presence of OAB and establishing treatment monitoring [18–20].

The aim of this prospectively designed study was to test PGE2 in OAB and its changes after BoNT-A therapy.

## Methods

### Patients

56 patients (48 female, 8 male, mean age of 63 years, range 18–87) with the typical symptoms of OAB were enrolled in this study (Group 1).

Overactive bladder specifically is defined as urgency, with or without urge incontinence, usually with frequency and nocturia [3]. So, all patients in the group of cases showed each of the symptoms with or without urge incontinence and have been in treatment accordingly. All patients were non-responder to medical treatment or discontinued anticholinergic therapy due to severe side effects. None of them underwent BoNT-A therapy before. The patients underwent a washout either by getting no more anticholinergic medication before treatment with BoNT-A, or terminated taking the medication after application of BoNT-A immediately. So until the full insertion of the BoNT-A effect after 14 days a sufficient wash out has taken place in any case.

In addition, none of the patients took medications with anticholinergic side effects in the investigated group.

31 healthy persons (19 female, 12 male, mean age of 59 years, range 21–72) with normal bladder function served as control group (Group 2). None of the control group showed lower urinary tract symptoms in the 14 days before as well on the day of blood collection.

Patient data are summarized in Table 1.

**Table 1:** **Summarized data of OAB patients (Group 1, Group 1A = wet OAB, Group 1B = dry OAB)) and control group (Group 2) (f = female, m = male, y = years)**

Group	Age (y, range)	Gender	Age (y, range)	OAB-Group	Gender	Age (range)
**1** (n = 56)	63 (18–87)	48 f	63.6 (18–87)	**1A** (n = 44)	40 f/4 m	63 (18–83)
		8 m	62 (57–80)	**1B** (n = 12)	8 f/4 m	64 (40–87)
**1 postop** (n = 30)	62.1 (40–83)	25 f	59.6 (40–83)	**1A** (n = 25)	21 f/4 m	63.6 (28–83)
		8 m	74.2 (67–80)	**1B** (n = 5)	4 f/1 m	60.4 (40–67)
**2** (n = 31)	59 (21–72)	19 f	62 (28–71)			
		12 m	56.3 (21–65)			

The study was approved by the ethical review committee of the Philipps University Marburg (AZ 12/11). Informed written consent was obtained from all participants before collecting blood samples for measurement.

### Inclusion criteria

Inclusion criteria were urodynamic proved non-neurogenic overactive bladder with detrusor hyperactivity or hypersensitive low capacity bladder without detrusor hyperactivity. Detrusorhyperactivity was diagnosed if a pattern of bladder muscle contraction was observed while urodynamics correlated to the symptoms of overactive bladder. On the other side hypersensitive low capacity bladder without detrusor hyperactivity was defined as the occurrence of early urgency before filling to 150 ml and a maximum bladder capacitiy less than 350 ml without pattern of bladder muscle contraction during urodynamics.

All patients with proven neurogenic bladder dysfunction, chronic pelvic pain, prostate hyperplasia (weight >30 gramm) or bladder outlet obstructions were not included in this study.

Evaluation included patient history, urine analysis, a voiding diary and urodynamic studies. Patients were only included into the control group if patient history and urine analysis were without pathological findings and “International Consultation on Incontinence Questionnaire – Short Form” (ICIQ-SF) and “Kings Health Questionnaire” (KHQ) scores showed no impaired quality of life.

All patients were investigated thoroughly and excluded if they did not meet the criteria.

### BoNT-A application

BoNT-A (500 MU Dysport^®^, Ipsen Pharma, Ettlingen, Germany) was injected into the detrusor muscle including trigone at 20 different locations in general or local anaesthesia under visual control.

#### Therapeutic success

Before and 4 weeks after treatment therapeutic success was assessed on the basis of standardized questionnaires ICIQ-SF and KHQ. Additionally, all patients were interviewed face-to-face about their individual quality of life, changes in number of used pads and leakage before and after BoNT-A treatment.

### Sample handling and PGE2 measurement

Before and 4 weeks after BoNT-A treatment, blood samples were collected in serum-gel-tubes and processed within 1 h. After centrifugation (3500 rpm for 10 min) serum samples were aliquoted and stored at -20°C. After defrosting, all samples were diluted (150 μL of sample +300 μL of Calibrator Diluent). Serum PGE2 (sPGE2) blood levels were measured at room temperature (18 – 23°C) by a commercialized enzyme linked immunosorbent assay (PGE2 Immunoassay, R&D Systems, Minneapolis, USA). Standard (25,000 pg/mL) was used to produce a dilution series (2500 pg/mL standard served as high standard, Calibrator Diluent as zero standard). The Microplate was prepared with Calibrator Diluent, zero standard and sample according to manufacturer’s instructions. After 1 h incubation at room temperature on a microplate shaker PGE2 Conjugate (50 μl) was added to each well, a 2 h incubation followed. Each well was washed with Wash Buffer (400 μl). Remaining Wash Buffer was aspirated and the plate blotted. Substrate Solution was added to each well (200 μl) and incubated for 30 minutes at room temperature. Stop Solution (100 μL) was added to each well. The optical density of each well was determined within 30 minutes, using a microplate reader set to 450 nm. Wavelength correction was set to 540 or 570 nm.

### Statistical analysis

The statistical data analysis was performed using the non-parametric Mann–Whitney U test and paired t-test to compare the different groups and the individual changes (IBM SPSS^®^ Statistics for Windows Version 22, Ehningen, Germany).

## Results

### General

Baseline data and sPGE2 levels were available for all 56 patients before BoNT-A therapy, hereof 44 with wet OAB (Group 1A), and 12 with dry OAB (Group 1B). Median Follow-up was 28 month (range 15–32). For 30 patients control visits 4 weeks after BoNT-A therapy were realized in order to assess the sPGE2 course in postoperative samples. In this group median follow-up was 30 month (range 15–32).

### Therapeutic success

OAB symptoms improved. This is reflected in significantly decreased urinary frequency (p < 0.01) and significant reduction of incontinence episodes (p < 0.05). Pads were needed significantly less frequent (4 (range 1–18) vs. 1(range 0–8), p < 0,05).

KHQ indicated a significant improvement of quality of life in all individual life situations (p < 0.05).

Mean duration of BoNT-A effect was 9 month (range 0–19). 3 patients (2 female, 1 male) reported no adequate effects after BoNT-A treatment and were ranked as non-responders.

### sPGE2 levels before BoNT-A

**Figure 1:**
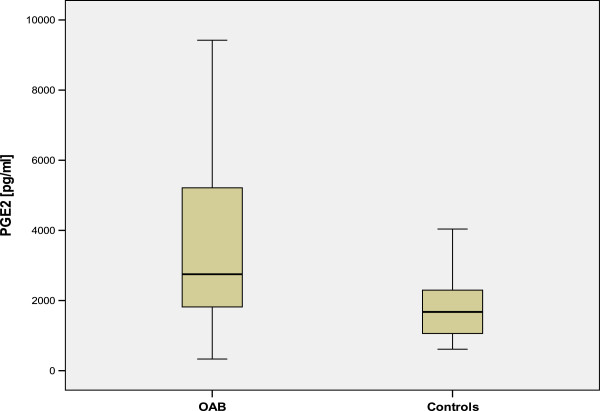
**sPGE2 levels in patients with OAB and control** ***group*** **(p < 0.05).**

The average concentration of sPGE2 in Group 1 was significantly higher (2749.5 pg/ml, SD 2375.7, range 332–9422) compared to Group 2 (1674.3 pg/ml SD 873, range 611–4038, p < 0.005, see Figure 1).

Subgroup analysis showed significantly increased sPGE2 levels in Group 1A compared to Group 1B (3241 pg/ml versus 1734 pg/ml, p < 0.01).

### sPGE2 levels after BoNT-A

**Figure 2:**
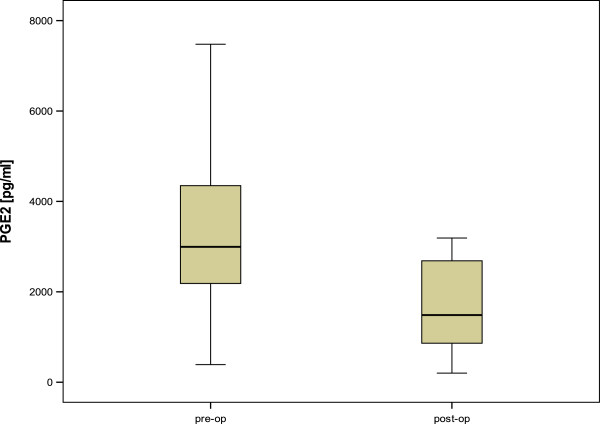
**sPGE2 levels before and 4 weeks after BoNT-A treatment (p < 0.005).**

Postoperative sPGE2 levels could be determined in 30 patients of Group 1. Compared to baseline, mean sPGE2 levels significantly decreased 4 weeks after BoNT-A therapy (2995 pg/ml, SD 2022, range 389–8861 versus 1486 pg/ml, SD 2414, range 201–11286; p <0.005, see Figure 2).

sPGE2 levels of the different groups are summarized in Table 2.

**Table 2:** **Summarized pre- and post-BoNT-A median sPGE2-levels of OAB patients (Group 1, Group 1A = wet OAB, Group 1B = dry OAB)) and control group (Group 2) (f = female, m = male, y = years)**

Group	sPGE2 pre [pg/ml]	Group	sPGE2 pre [pg/ml]	Group	sPGE2 [pg/ml]
**1** (n = 56)	2749.5 (332–9422)	**1A** (n = 44)	3241 (389–9422)	**1** (n = 30)	***pre:*** 2995 (389–8861)
		**1B** (n = 12)	1734 332–8861)		***post:*** 1486 (201–11286)
**2** (n = 31)	1674.3 (611–4038)				
**p-value**	< 0.005		< 0.01		< 0.005

Decrease of sPGE2 levels was correlated with the mean duration of drug effect (9 month). The subgroup with a shorter drug effect <9 month presented a significantly lower sPGE2 decrease (22.2%) compared to patients with a drug effect >9 month (57.4%, p < 0.05).

Additionally, in 2 of 3 non-responders (66.6%) sPGE2 levels increased after treatment up to 51% compared to baseline (Pat 1: 1815 pg/ml to 2749 pg/ml (+51.5%)/Pat 2: 1797 pg/ml to 2333 pg/ml (+29.8%)).

**Figure 3:**
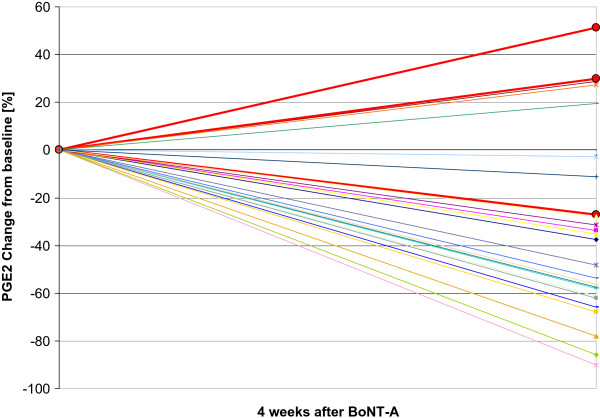
**Percentage decrease (-)/increase (+) of sPGE2 in 30 patients 4 weeks post BoNT-A treatment (red lines: non responders to BoNT-A).**

Percental decrease/increase of each patient is illustrated in Figure 3.

In the study period 6 patients (5 female, 1 male, mean age of 63.7 years, range 40–76) re-visited our department due to progressively increasing urgency and frequency as the BoNT–A effects wore off after a median time of 8.5 month (range 6–12). Measurements confirmed a re-rise of sPGE2 in all patients after initial sPGE2 decrease. After reapplication of BoNT-A a re-decrease of sPGE 2 could be detected.

## Discussion

OAB represents a rising health problem massively affecting quality of life. Intravesical BoNT–A administration has been shown to be a safe and effective therapy for refractory idiopathic OAB. It is a reasonable treatment of patients impossible to take anticholinergic medication [15–17, 21, 22]. To date several studies tried to elucidate cause and mechanism of OAB, but it is still poorly understood [4, 18, 19, 23]. Up to now a marker for OAB is lacking. In general a biomarker should be able to indicate and define both the presence and severity of a disease as well as progression/relapse and response on therapy [20]. Prostanoids are inflammatory mediators induced by mitogens or proinflammatory agents. PGE2 associated physiological responses are found in the whole human system. Typical prostanoid-induced actions are smooth muscle relaxation and contraction and neuromodulation (inhibition or release of neurotransmitters, regulation of inflammatory mediation, thermoregulation or sleep induction). In the urinary bladder, PGE2 is released from the urothelium, smooth muscle [24, 25], glial cells and neurons as required [26, 27]. Our results clearly demonstrate the relationship between PGE2 and OAB reflected in significantly elevated serum levels of PGE2 in patients suffering from OAB. It is particularly noticeable that there is a wide range of sPGE2 levels with pronounced inter-individual differences. These findings are in line with studies trying to find a urinary marker to differentiate OAB, DO or interstitial cystitis (IC)/bladder pain syndrome (BPS). Recent studies showed significantly increased urinary PGE2 in male and female OAB patients, underlining the important but still unclear role of PGE2 [5, 8, 28]. Moreover, our data detect a correlation of sPGE2 levels with the severity of OAB reflected by significant higher levels in wet compared to dry OAB. This possible potential to differentiate between vairous OAB stages reveals PGE2 as promising for future use as a marker and additional helpful tool for therapeutic decision in OAB.

For the first time we present data that sPGE2 development is influenced by intravesical BoNT-A therapy in OAB. We found a significant decrease of sPGE2 levels 4 weeks after BoNT-A application. Furthermore, we detected a correlation between the success of therapy as well as the degree and duration of decreased sPGE2 levels. A shorter drug effect (<9 month) was significantly associated with less sPGE decrease. Non-responders even displayed a sPGE2 increase or only a slight decrease. A recent study examined PGE2 urine levels in female OAB patients treated with anticholinergics and no significant changes of PGE2 urine levels could be detected after 4 weeks of treatment [28]. These differences in PGE2 detection in urine and serum may be due to the different methods and body fluids used and due to the diverging administered therapeutic substances. But how far are local urinary levels of the inflammatory mediators relevant to the inflammatory process at all? In particular, anticholinergics and BoNT-A are acting by different receptor mediated signal transduction pathways affecting Ca^++^ influx with impact on PGE2 release [29, 30]. The regulation and action of prostaglandins in the urinary bladder are still obscure. In addition, we verified a re-rise of sPGE2 when BoNT-A effects wore off and OAB relapsed. A limitation of this prospective single-center examination was the relatively small patient cohort of 58 OAB-patients. These first promising results, that show the potential of sPGE2 as a biomarker in OAB, have to be confirmed in further investigations with a larger population.

## Conclusions

These promising first data are indicate the helpfulness of sPGE2 in OAB. sPGE2 seems to have the potential as a biomarker for OAB defining the presence, severity and response to therapy and relapse. Future investigations have to evaluate the relevance of sPGE2 to diagnose and monitor patients with OAB. BoNT-A treated patients could be monitored using sPGE2 as an early marker to anticipate decreasing effects of BoNT–A in combination with a voiding diary. Regarding the non-responder group, an efficient monitoring could filter these patients and lead to an alternative treatment. To what extent sPGE2 can be useful as such needs to be examined in further investigations.

### Consent

Written informed consent was obtained from the patient for the publication of this report and any accompanying images.
